# Dynamics and thermal stability of the bypass polymerase, DinB homolog (Dbh)

**DOI:** 10.3389/fmolb.2024.1364068

**Published:** 2024-04-30

**Authors:** Jenaro Soto, Sean L. Moro, Melanie J. Cocco

**Affiliations:** ^1^ Department of Pharmaceutical Sciences, University of California, Irvine, CA, United States; ^2^ Department of Molecular Biology and Biochemistry, University of California, Irvine, CA, United States

**Keywords:** Dbh, Y-family polymerase, DinB homolog, NMR, thermal stability, dynamics, cold denaturation

## Abstract

The DinB homolog polymerase (Dbh) is a member of the Y-family of translesion DNA polymerases that can synthesize using a damaged DNA template. Since Dbh comes from the thermophilic archaeon *Sulfolobus acidocaldarius,* it is capable of functioning over a wide range of temperatures. Existing X-ray structures were determined at temperatures where the protein is least active. Here we use NMR and circular dichroism to understand how the structure and dynamics of Dbh are affected by temperature (2°C–65°C) and metal ion binding in solution. We measured hydrogen exchange protection factors, temperature coefficients, and chemical shift perturbations with and without magnesium and manganese. We report on regions of the protein that become more dynamic as the temperature is increased toward the functional temperature. Hydrogen exchange protection factors and temperature coefficients reveal that both the thumb and finger domains are very dynamic relative to the palm and little-finger (LF) domains. These trends remain true at high temperature with dynamics increasing as temperatures increase from 35°C to 50°C. Notably, NMR spectra show that the Dbh tertiary structure cold denatures beginning at 25°C and increases in denaturation as the temperature is lowered to 5°C with little change observed by CD. Above 35°C, chemical shift perturbation analysis in the presence and absence of magnesium and manganese reveals three ion binding sites, without DNA bound. In contrast, these bound metals are not apparent in any Dbh crystal structures of the protein without DNA. Two ion binding sites are confirmed to be near the active site, as reported in other Y-family polymerases, and we report a novel ion binding site in the LF domain. Thus, the solution-state structure of the Dbh polymerase is distinct from that of the solid-state structures and shows an unusually high cold denaturation temperature.

## 1 Introduction

DinB homolog polymerase (Dbh) is produced by the thermophilic archaeon *Sulfolobus acidocaldarius* (*Sulfolobus acidocaldarius*), that grows optimally at temperatures close to 75°C–80°C and pH values between 2–4 (Grogan, 1989; Rastädter et al., 2021). Consequently, it is important to understand the effect of temperature on the structure of this enzyme. To adapt to extremely acidic environments, extremophiles possess efficient mechanisms to maintain cellular homeostasis, such as proton pumps (Baker-Austin and Dopson, 2007). However, microorganisms do not have the ability to regulate cell temperature; consequently, they require proteins adapted to function in extremely high temperatures. This thermal stability is apparent in Dbh which has been shown to remain structurally and functionally sound up to 65°C (Potapova et al., 2002).

The function and efficiency of Dbh are known to be modulated by changes in temperature. This was clearly revealed in a primer extension study that showed Dbh efficiency increasing as the temperature was increased from 22°C to 65°C (Potapova et al., 2002). Furthermore, *in vivo* studies conducted under physiological temperatures (70°C–80°C) for *S. acidocaldarius* Dbh reveal that the enzyme imposes a three base-pair frameshift on triple repeats of Sacp*yrE* and Sso*pyrE* which has never been observed in *in vitro* studies (Sakofsky et al., 2012) that were not performed at physiological conditions. Since the activity of Dbh is largely dependent on temperature changes we set out to understand how temperature influences Dbh dynamics, secondary, and tertiary structures. In addition, we explored how metal ions associated with Dbh in solution and in the absence of DNA.

**FIGURE 1:**
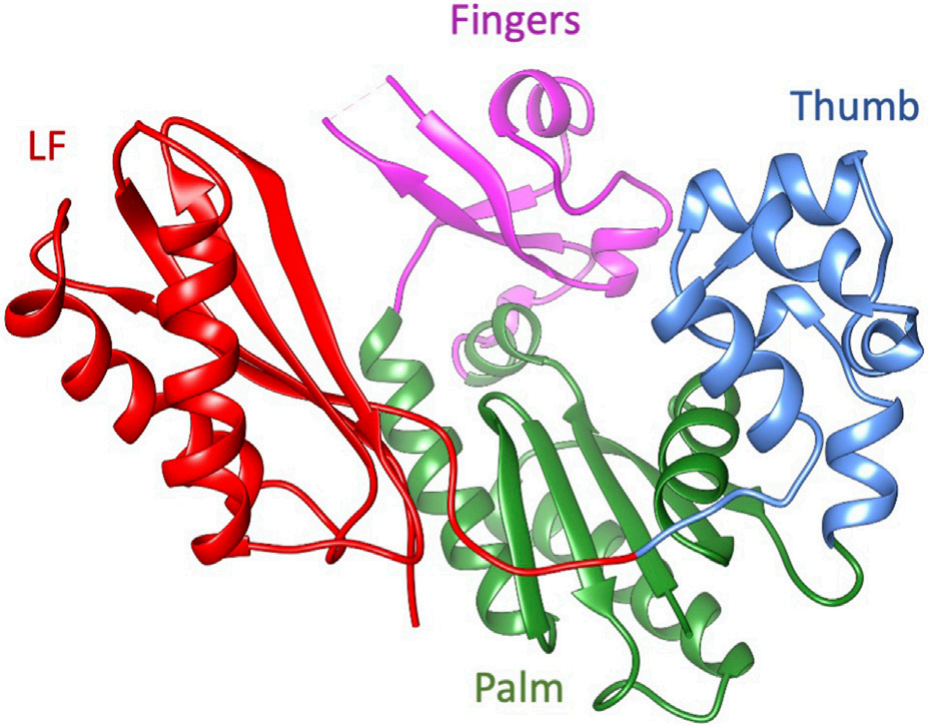
The ribbon diagram of Dbh polymerase (PDB: 1K1S). Fingers domain (residue 20–35, 39–77: magenta), thumb domain (residue 172–236; cornflower blue), palm domain (1–19, 78–171; green), and little finger (LF) (residues 237–344; red).

Dbh is a Y-family polymerase; this group of polymerases perform translesion synthesis, with low fidelity and low processivity. Y-family polymerases can synthesize across damaged DNA templates but with high error rates limited by fast off-rates (Pata, 2010). Compared to other Y-family polymerases, Dbh replicates undamaged DNA with an error rate that is 10—100 fold higher (Johnson et al., 2000a; Johnson et al., 2000b; Ohashi et al., 2000; Tang et al., 2000). Despite being phylogenetically unrelated to other families of polymerase, the Y-family structure can be likened to a right-hand conformation found in most polymerases, with the addition of a unique N-terminal domain referred to as the little finger (LF), wrist or other various names (discussed below) (Figure 1). Polymerases are complex molecular machinery highly dependent on dynamics to perform their functions that include binding substrates, synthesis, and translocation. Although the three-dimensional structure has been studied by several groups, the dynamics of Dbh have not been thoroughly explored.

The fact that Dbh crystallizes readily has facilitated the study of several static structures (Silvian et al., 2001; Zhou et al., 2001; Wilson and Pata, 2008) and biochemical characterizations (Boudsocq et al., 2004; Wilson and Pata, 2008; Wang et al., 2022). Initial Dbh structures were reported to derive from *Sulfolobus solfataricus* (now *Saccharolobus solfataricu*s (Sakai and Kurosawa, 2018)), however it was discovered through genomic sequencing that they belong to *S. acidocaldarius* (Kulaeva et al., 1996; Ohmori et al., 2001; Boudsocq et al., 2004). To prevent future confusion the Dbh protein sequence studied here is identical to UniProt accessions P96022 and Q4JB80 (UniProt, 2023). This protein sequence contains one modification: cysteine to serine mutation at residue 31 to facilitate protein study at high concentrations without the risk of dimerization. The entirety of the work presented here uses the mutated C31S form of Dbh. Crystal structures revealed that Dbh is composed of a catalytic core that includes the finger domain, palm domain, and thumb domain (Figure 1). In addition, the core structure is connected to the LF by an unstructured linker. This domain has been referred to by many names: it has been described as the LF in archaeal and bacterial enzymes (Ling et al., 2001) and the wrist domain (Silvian et al., 2001), or Polymerase-associated domain (PDA) in eukaryotic Y-family polymerases (Trincao et al., 2001). For the remainder of this article this domain will be referred to as LF.

At this time, seven crystal structures of Dbh exist with only one study showing Dbh interacting with a metal ion (calcium) bound to the active site (Wilson and Pata, 2008). However, multiple related Y-family polymerases have been solved with two magnesium (Mg^2+^) ions in the active site (Irimia et al., 2010; Pata, 2010). Notably these ions are always present when the structures are solved with DNA. As far as we know, Dbh has not been shown previously to bind multiple ions in the absence of DNA. Moreover, despite the detailed structure data available on Dbh we still lack a good understanding of the dynamics and flexibility within the protein.

Changes in temperature can have a large effect on the dynamics and function of Dbh. For example, it has been shown that changes in temperature affect the rate of opening and closing of other thermally stable polymerases such as *Thermus aquaticus* DNA polymerase (Turvey et al., 2022). In addition, we know that Dbh function is enhanced by increasing temperature (Potapova et al., 2002). To truly understand Dbh dynamics we must first study it alone, in solution, and over a wide range of temperatures. To determine how Dbh dynamics are affected by temperature we used solution-state NMR spectroscopy and circular dichroism (CD) over a range of temperatures (2°C–65°C). Notably, we find evidence for cold denaturation of Dbh below 25°C. We also used chemical shift mapping to study the effect of Mg^2+^ and Mn^2+^ in the absence of DNA.

## 2 Materials and methods

### 2.1 Protein expression

The Dbh gene was incorporated into the vector pKKT7-H (a derivative of pKK233, Promega) containing an N-terminal His6 tag (MHHHHHHLVPRGM). Quick-change mutagenesis (Stratagene) was used to change Cys31 to Ser to eliminate potential formation of disulfide bonds. Transfected *E. coli* BL21 cells were grown in 1L Neidhart’s minimal media (Neidhardt et al., 1974) at 37°C containing 1 g ^15^N ammonium chloride; expression was induced by the addition of 1 mM IPTG. Protein was expressed for 5 h; subsequently, the cells were harvested by centrifugation and frozen at −80°C. Dbh was purified from cell lysate by Ni-NTA affinity chromatography under native conditions, and then dialyzed into buffer (20 mM HEPES, 100 mM NaCl, 50 μM EDTA, 50 μM NaN_3_, pH 7.5) at 4°C, followed by one change of buffer without EDTA (NMR buffer = 20 mM HEPES, 100 mM NaCl, 50 μM NaN_3_, pH 7.5). To prepare the NMR samples, Dbh protein was concentrated to between 0.3–0.5 mM and transferred into a 5-mm Shigemi tube. D_2_O was added to the sample for a final concentration of 10% v/v.

### 2.2 NMR experiments

*Hydrogen exchange:* Samples of ^15^N-labeled Dbh (0.5 mM or higher concentration) were transferred into deuterated NMR buffer (20 mM HEPES, 100 mM NaCl, 50 μM NaN_3_, pD 7.5), using a P10 desalting column equilibrated with the deuterated buffer. The sample was transferred to a Shigemi NMR tube and immediately placed in an 800 MHz Varian Inova NMR spectrometer, containing an xyz triple resonance probe, equilibrated at 35°C or 50°C. After shimming and tuning the magnet, the acquisition of the first 15N-1H TROSY-HSQC (Pervushin et al., 1997) spectrum was started approximately 15 min after insertion of the protein sample into the magnet. Additional ^15^N-HSQC spectra were measured sequentially every 147 min and 13 s (2.454 h), except for the last five spectra at 50°C, for which additional scans were taken (2x for the 17th through 20th spectra, 4x for the 21st spectrum) to improve the signal-to-noise ratio. A total of 20 spectra were collected at 35 °C and 50 °C. Due to the additional length of acquisition in the last five spectra at 50°C, the acquisition of the 18th, 19th, 20th, and 21st spectra was started 4.907 h after the start of the previous spectra. Assigned spectra for initial time points at both temperatures are shown in (Supplementary Figure S1). The data were processed using NMRPipe (Delaglio et al., 1995) and analyzed using CCPNMR Analysis (Vranken et al., 2005) (RRID:SCR_016984). The peak intensity was plotted as a function of time and fit to a single-order exponential-decay function (I(t) = I_0_ × e^−kt^) to extract the exchange rate. Representative time points and rate fits to the experimental data are displayed in (Supplementary Figure S2). To obtain the protection factors the hypothetical exchange rate for the amide proton in a random coil conformation was calculated, corrected for the effect of side chain identity to the left and right of the amide proton according to (Molday et al., 1972; Bai et al., 1994).

*Temperature Coefficients (TC):* Conventional ^15^N-HSQC spectra were taken at 35°C, 45°C, 50°C, 55°C, and 65°C, on samples containing at least 0.5 mM ^15^N-labeled Dbh in 20 mM HEPES, 50 mM NaCl, 50 μM EDTA, pH 7.5, with 10% (v/v) D_2_O. The data were processed with NMRpipe (Delaglio et al., 1995), and visualized using CCPNMR Analysis 2.5 (RRID:SCR_016984, (Vranken et al., 2005). For each residue, H chemical shift (ppm) was plotted as a function of temperature (K) and fit to a straight line. The slope*1000 for each fit was used as TC for each residue. The standard error of the slope was used as TC uncertainty. Only fits with *R* ^2^ > 0.75 were used. Non-linear fits were not used, a total of 10 non-linear TC values were discarded. The TC average for each domain only includes amides located in secondary structure regions, and excluded amides in loop regions and amides that are exposed to solvent based on the 1K1S crystal structure.

*Ion chemical shift perturbations:* All samples were made with 20 mM HEPES, 100 mM NaCl, 50 μM NaN_3_, at pH 7.5. The Mg^2+^ sample contained 5 mM MgCl_2_. The Mn^2+^ sample contained 5 mM of MnCl_2_. Data were analyzed with CCPNMR Analysis (Vranken et al., 2005) (RRID:SCR_016984).

### 2.3 Circular dichroism

200 μL of 0.28 mg/mL Dbh protein in buffered solution (50 mM sodium phosphate, 100 mM NaCl, pH 7.5) were transferred into a CD cuvette with a 1-mm path length. The cuvette was placed in the sample cell Jasco J-810 spectropolarimeter equipped with a Peltier temperature controller, and the cell was calibrated at 35°C. A spectrum was collected at 35°C from 260 nm to 185 nm, with a scan rate of 50 nm/min, data points were collected every 0.5 nm and 10 total scans. The cell was then cooled at a rate of 0.5°C/min to 7°C, with the ellipticity value measured at 208 nm every 2°C, then after a full spectrum was taken at 7°C with the same parameters as above. The sample was then removed from the CD cuvette, the cuvette was cleaned, and a second 200 μL 0.28 mg/mL Dbh protein sample was transferred into the cuvette. The cuvette was placed into the cell equilibrated at 35°C, another full CD spectrum was taken at this temperature, and the sample was heated to 65°C at a rate of 0.5°C/min, with the ellipticity value at 208 nm taken every 2°C. One full spectrum was then taken at 65°C. After adjustments to the chiller unit, a third 0.28 mg/mL Dbh sample was used to collect full CD spectra again at 7°C and then at 2°C.

### 2.4 Dynamic light scattering

Protein samples used for NMR measurements were diluted with the NMR buffer solution (50 mM sodium phosphate, 100 mM NaCl, pH 7.5). The protein concentration was then measured by light absorbance at 280 nm and determined to be 1.1—1.3 mg/mL. The protein solution was pipetted into a disposable plastic cuvette with a path length of 1 cm and placed in a Malvern Zetasizer Nano DLS instrument. The temperature was equilibrated at 35°C and measurements were taken for 30 s and repeated ten times. The temperature was then lowered to 5°C, allowed to equilibrate for 10 min at 5°C, then measurements were taken as above.

### 2.5 Protein visualization

We used UCSF Chimera to visualize Dbh (Pettersen et al., 2004). Structure 1K1S was used for all analyses (Silvian et al., 2001). The addition of hydrogens and solvent accessibility were obtained using Chimera default settings.

## 3 Results

### 3.1 Structural stability of Dbh from 35°C to 65°C

**FIGURE 2:**
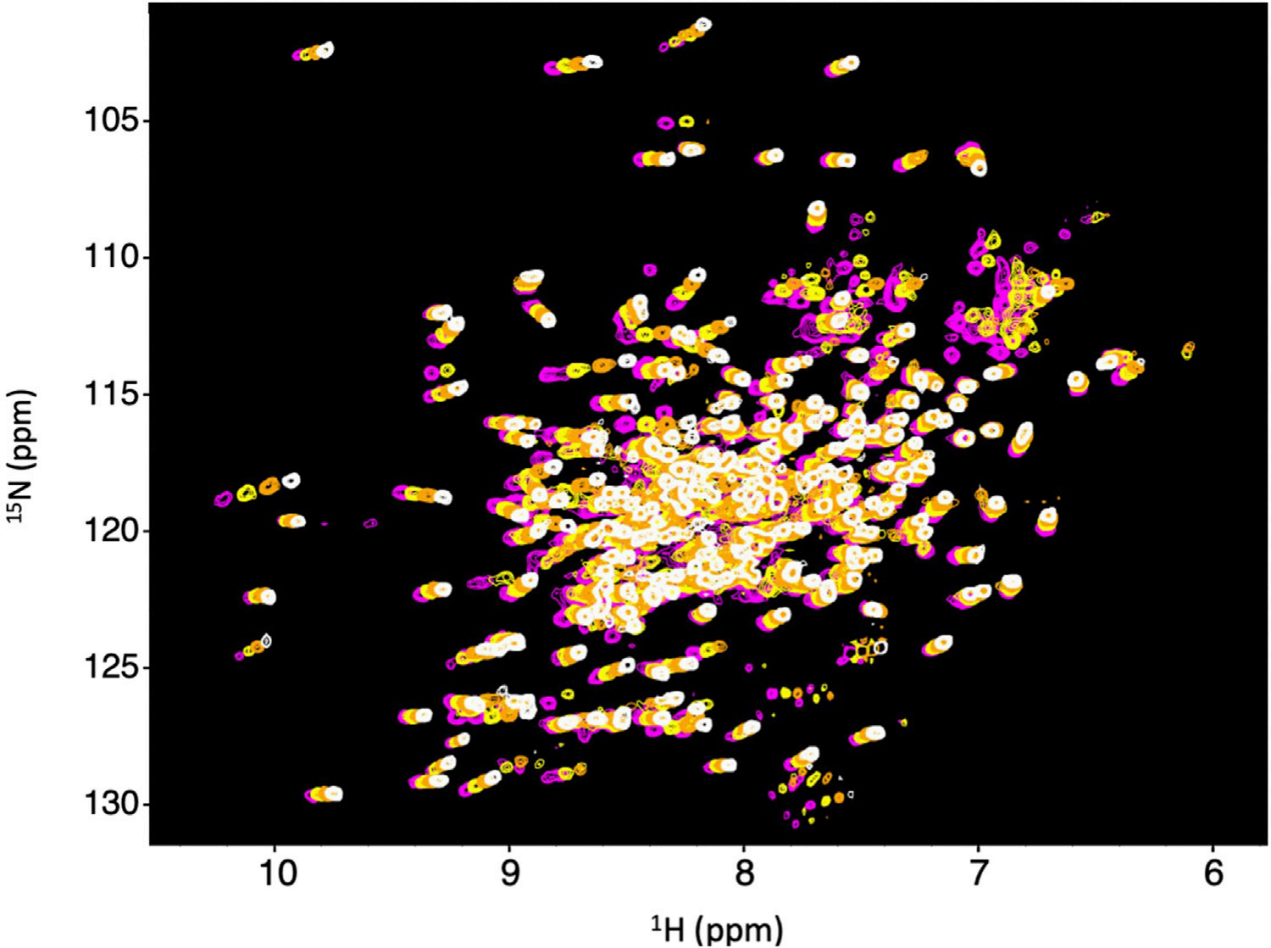
Dbh is structurally stable from 35–65^°^C. ^1^N–^15^N HSQC of Dbh at 35^°^C (magenta), 45^°^C (yellow), 55^°^C (orange), 65^°^C (white). Spectra do not exhibit drastic changes over this range of temperatures. The consistent, well-dispersed spectra tell us that Dbh remains structurally similar across this range of temperatures. The upfield shifting of ^1^H peaks (*X*-axis) is normally with increasing temperature. We analyze the extent of the shift (Temperature Coefficient, TC) to determine unfolding of Dbh (Table 1).

We obtained ^15^N-HSQC NMR spectra to determine if Dbh maintains tertiary structure between 35°C and 65°C. The spectra were collected at 35°C, 45°C, 55°C, and 65°C. All spectra remained well dispersed and relatively similar across this range of temperatures (Figure 2). We did observe the normal phenomena of temperature dependent chemical shift changes as temperature increases (Ohnishi and Urry, 1969). We used this temperature dependent chemical shift to calculate TC (discussed later) to determine sensitivity to temperature changes. The retention of well dispersed spectra at temperatures ranging between 35°C–65°C informs us that Dbh retains a well folded and consistent tertiary structure throughout this range of temperatures.

### 3.2 Cold denaturation

**FIGURE 3:**
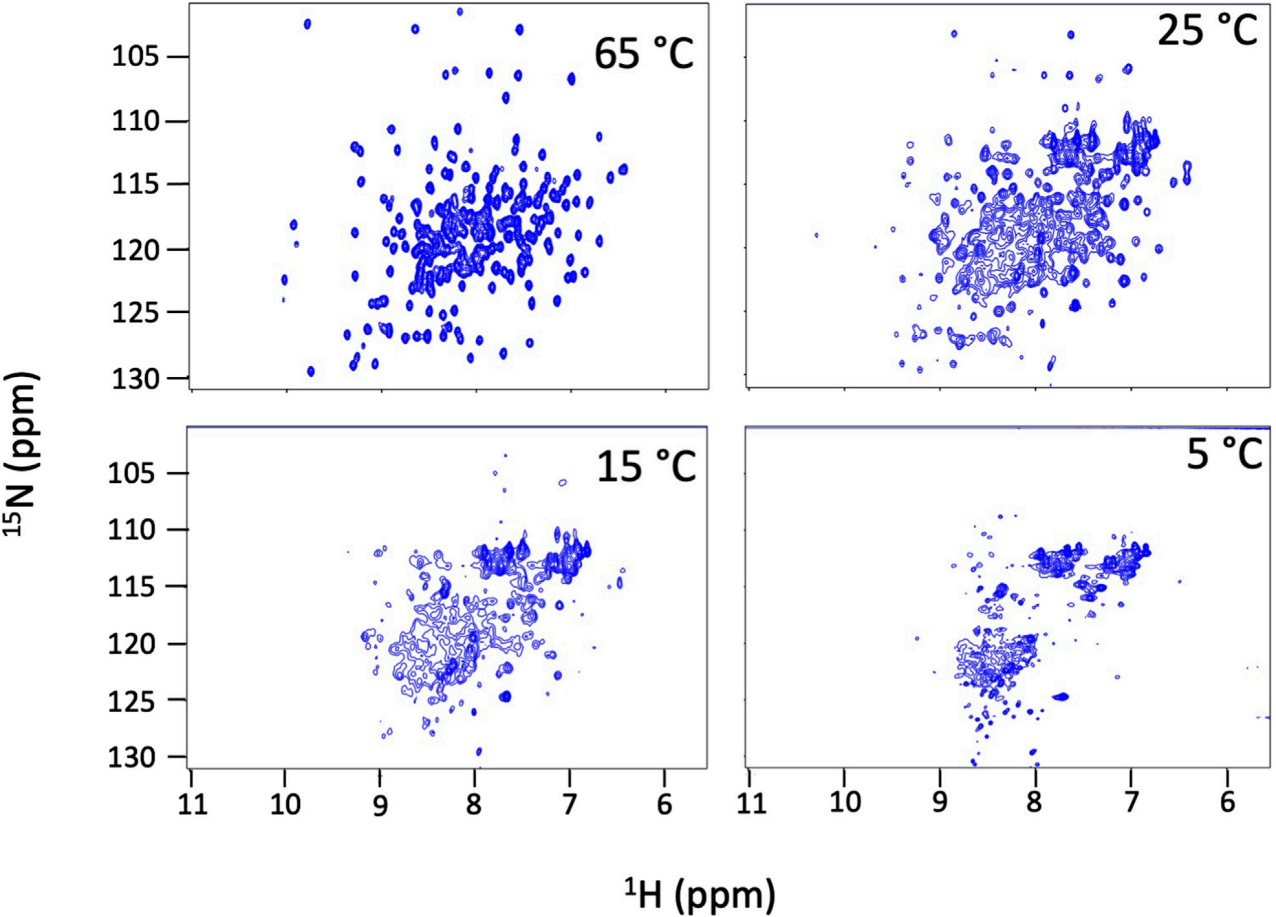
Dbh cold denatures. NMR ^15^N-HSQC spectra of Dbh at 65°C, 25°C, 15°C and 5^°^C. Dispersed peaks of the structured protein broaden and shift to the region where random coil signals resonate (8–9 ^1^H ppm) at cold temperatures indicating a loss of tertiary structure. These spectral changes are fully reversible and the protein remains soluble and monomeric.

**FIGURE 4:**
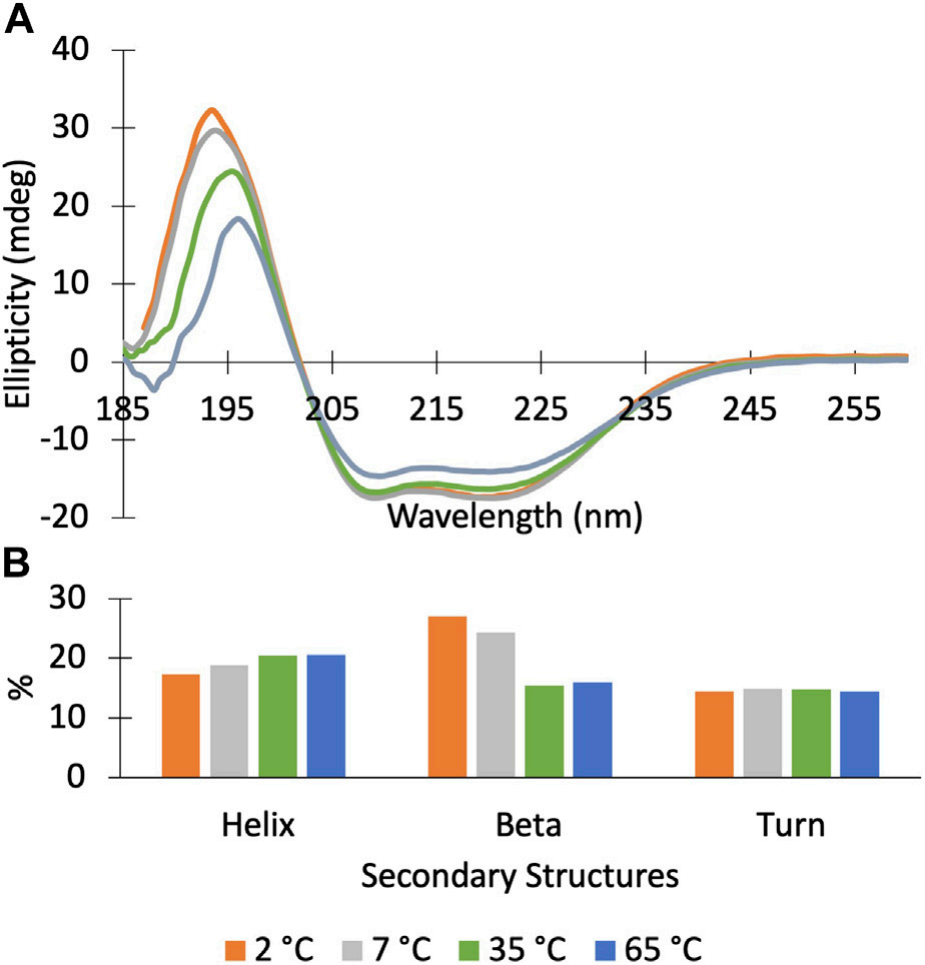
Circular Dichroism spectra of Dbh polymerase show that it retains secondary structure at temperatures ranging from 2–65°C. **(A)** CD scans (185–260 nm) of DBH at 2^°^C (orange), 7^°^C (grey), 35^°^C (green), 65^°^C (blue). **(B)** Secondary structure deconvolution via Beta Structure Selection (BeStSeL) (Micsonai et al., 2018) showed that there is a slight decrease in percent helicity and an increase in percent beta strand as temperature is decreased.

We find that Dbh undergoes cold denaturation of tertiary structure below 25 °C**.** Cold denaturation is a phenomenon that all proteins are predicted to experience, however, it is extremely rare for it to occur above the freezing point of water (0 °C) (Privalov, 1990). We collected ^15^N-HSQC spectra at cold temperatures ranging from 5°C to 25°C. These spectra show a reversible decrease in tertiary structure starting at 25 °C which progresses as temperatures get colder (15°C, 5°C) (Figure 3). To determine if this is a complete loss of structure, we assessed the secondary structure content using CD spectroscopy. Figure 4 shows CD spectra for Dbh at 2°C, 7°C, 35°C, and 65°C. In contrast to the NMR results, CD revealed that the secondary structure of Dbh is consistent over this temperature range, similar to what has been reported for Dpo4 (Sherrer et al., 2012). We used Beta Structure Selection (BeStSeL) (Micsonai et al., 2018) to deconvolute the CD spectra; our assessment of secondary structure content shows only a slight decrease in percent helicity and a small increase in percent beta strand as temperature decreases (Figure 4). The protein is monomeric with a diameter between 6–7 nm at both 5°C and 35°C as determined by dynamic light scattering (DLS), shown in Supplementary Figure S3. Collectively, these results are consistent with a molten globule unfolded state at cold temperatures.

### 3.3 Effect of temperature on hydrogen bond stability

Dbh dynamics were probed by measuring amide HX rates (*k*_*ex*_) and calculating protection factors at each temperature. We previously published NMR peak assignments for Dbh at these two temperatures (Moro and Cocco, 2015). Amide hydrogens that are readily exposed to solvent will exchange at a higher rate compared to hydrogens that are involved in secondary structure or sequestered away from solvent (*e*.*g.,* buried in a hydrophobic core).

We determined *k*_*ex*_ by measuring peak volumes as a function of time after transfer into D_2_O and fit the results to calculate the exchange rate (Supplementary Figure S2). PFs were calculated by *k*_*rc*_ */k*_*ex*_, where *k*_*rc*_ is the exchange rate of a backbone amide in a random coil and *k*_*ex*_ is the experimental exchange rate fit from the NMR peak volumes. *k*_*rc*_ was calculated based on in Bai, et al. (Bai et al., 1994). Obtaining PFs from multiple amides in each domain gives us a thorough understanding of the dynamics throughout the Dbh structure. Our Dbh construct is composed of 353 residues, 15 of which are prolines that do not have a corresponding amide hydrogen. In addition, Dbh has four distinct small domains with significant solvent accessible area. Consequently, many amides exchanged immediately upon transfer into D_2_O. At 35°C we were able to observe signals of 82 residues that remain in D_2_O. Eighteen residues exchanged too fast to obtain sufficient points to calculate an exchange rate, and 26 remained unchanged (stable) throughout the experiment (Supplementary Table S1). In contrast, at 50°C we obtained HX information on 43 residues. Seven of these exchanged too fast to obtain sufficient data to reliably fit exchange rates and 21 remained unchanged (stable) throughout the experiment (Supplementary Table S1).

**FIGURE 5:**
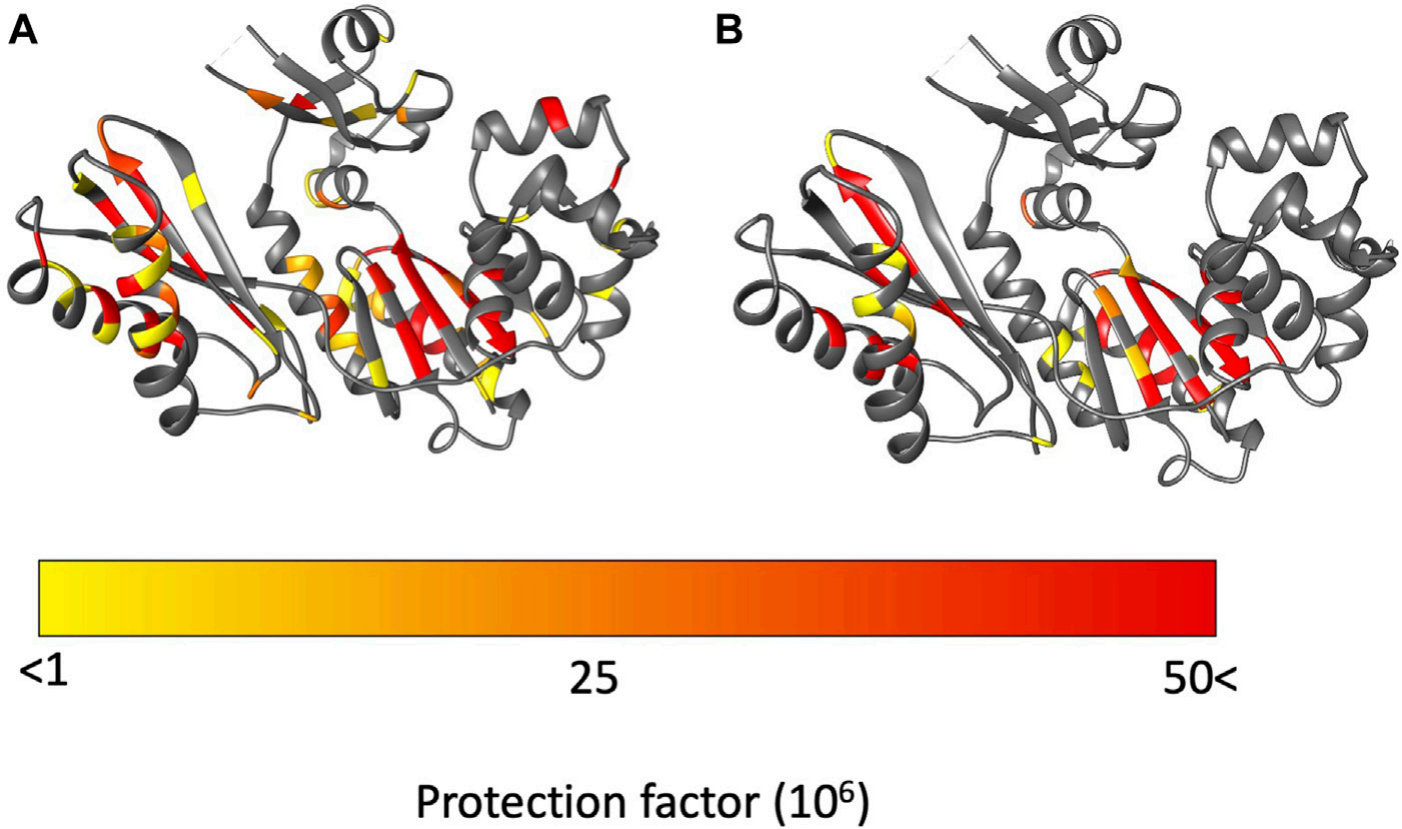
Protection factors (PFs) mapped on to Dbh crystal structure (PDB: 1K1S). **(A)** Dbh PFs at 35^°^C. **(B)** Dbh PFs at 50^°^C. Highly protected amides that remained stable throughout the hydrogen exchange experiment are colored in red. In contrast, amides with signals present initially but exchange too fast to calculate an exchange rate are colored in yellow.

The palm domain is composed of residues 1–19, 78–171. This is a critical domain because it holds the active site for nucleotide extension. At 35°C we observe protection for residues in the beta-sheet and two alpha helices that comprise the palm domain (Figure 5). We observe a minor decrease in protection at 50 °C. Much of the decrease in protection is in proximity to the major helices in the palm domain, which include helices E and F and strand 6. In this region of the palm domain we observe seven residues that lose complete protection (84, 85, 88, 91, 136–138) and five residues lose over 70% of their protection (89, 92, 106, 108, 124, 135) with the increase in temperature.

The fingers domain is composed of residues 20–77. The protection factor data shows this domain is more dynamic at 50 °C than at 35°C. At 35°C signals from two residues (20, 68) are present immediately after transfer to D_2_O but exchange too rapidly to determine exchange rates/PFs. The remaining seven observable residues (19, 28, 30, 33 50, 55, 74) are stable enough to obtain an exchange rates and protection factors (Figure 5; Supplementary Table S1). Two of the residues (50, 74) have relatively high PFs which indicate resistance to local unfolding at 35°C. Both residues are involved in secondary structure hydrogen bonding. Solvent accessibility surface area (SASA) calculations showed these amides are not exposed to solution. These residues are found 12.9 Å away from each other which indicates far reaching stability of the domain at 35 °C. In contrast, at 50°C all residues in the finger domain exchange too fast to measure. These two HX experiments reveal that the fingers domain of Dbh is significantly more dynamic at 50°C than at 35°C.

The thumb domain is composed of residues 172–236. At 35°C the thumb domain contains six observable residues (175, 178, 184, 194, 201, 206) at the start of the HX experiments. Two residues (194, 201) have high protection factors at 35°C. Residue 194 is involved in a helical section of the protein and residue 201 is in an unstructured part of Dbh between two helices. Both residues 194 and 201 amides have no exposure to solvent (SASA = 0 Å).

The LF domain is composed of residues 237–344 and becomes slightly more dynamic at 50 °C than at 35 °C. Two residues (287, 290) were extremely stable (no exchange) throughout the experiment at 35 °C. The only regions that showed a uniform decrease in protection in the LF were helix N (258–276) and helix O (307–324). All other decreases in protection factors were evenly distributed throughout the LF domain (Figure 5).

### 3.4 Temperature coefficients reveal local unfolding

Temperature coefficients are calculated from the chemical shift change with temperature. It has been shown that amides not involved in intramolecular hydrogen bonding (exposed to solvent) and amides that unfold in response to temperature changes have TC values below −4.6 ppb/K (Baxter and Williamson, 1997; Cierpicki and Otlewski, 2001; Cierpicki et al., 2002). We implemented TC analysis and obtained values that agreed with the crystal structure. We see that most residues with TC values below −4.6 ppb/K belong to amides that are not involved in hydrogen bonding and TC values more positive than −4.6 ppb/K belonged to hydrogen bonding amides in secondary structure (Supplementary Figure S4; Table 1). However, we did find several exceptions; for example, helix E (residue 77–94) of the palm domain gives TC values more negative than −4.6 ppb/K. This includes residues 82, 86, and 89 all of which are involved in hydrogen bonding and have no solvent exposure based on the crystal structure 1K1S. These data indicate that this particular region of the palm domain is sensitive to increases in temperature with unfolding and increasing exposure to solvent. Helix F of the palm domain also shows this unfolding (amide exposure to solvent) with temperature. In the thumb domain, although the crystal structure shows that residue 176 is involved in an intramolecular hydrogen bond within a helix, its TC value corresponds to an amide that also becomes exposed to solvent (<-4.6 ppb/K) as the temperature is raised.

**TABLE 1:** TC averaged across the entire domain, and across secondary structures are given. Average excludes amides in loop regions, amides not involved in hydrogen bonds, and amides exposed to solvent. Specific TCs for each residue are provided in Supplementary Table S1. Structural analysis based on the Dbh crystal structure 1K1S. Cells denoted with this symbol (**) did not have three or more TC values so an average could not be calculated. Averaging TC values across secondary structures and domains can give a measure of local stability. Dynamic regions have a TC average value that is more negative compared to that of rigid regions.

TC average by domain	
Domain	TC average (ppb/K)
Finger domain (20–77)	−3.19
Palm domain (1–19, 78–171)	−2.77
Thumb domain (172–236)	−3.28
LF domain (246–344)	−2.69
**TC average by secondary structure**	
**Helix**	
A (10–20)	−1.62
B (21–24)	**
C (47–52)	**
D (60–66)	−2.84
E (77-94)	−2.61
F (121–138)	−2.58
G (148–158)	−2.88
H (171–178)	−2.95
I (180–188)	−3.96
J (189–198)	−3.14
K (202–209)	−3.07
L (210–219)	−3.51
M (220–230)	**
N (258–276)	−2.21
O (307–324)	−2.59
**Strands**	
1 (3–8)	−2.58
2 (28–33)	−2.91
3 (41–45)	−2.84
4 (72–74)	**
5 (98–103)	−2.58
6 (106–110)	−3.43
7 (141–147)	−2.4
8 (164–166)	**
9 (241, 242)	**
10 (246–256)	**
11 (283–290)	−1.99
12 (295–301)	**
13 (331–339)	−3.74
14 (341,342)	**

### 3.5 Divalent ion binding in the absence of DNA

**FIGURE 6:**
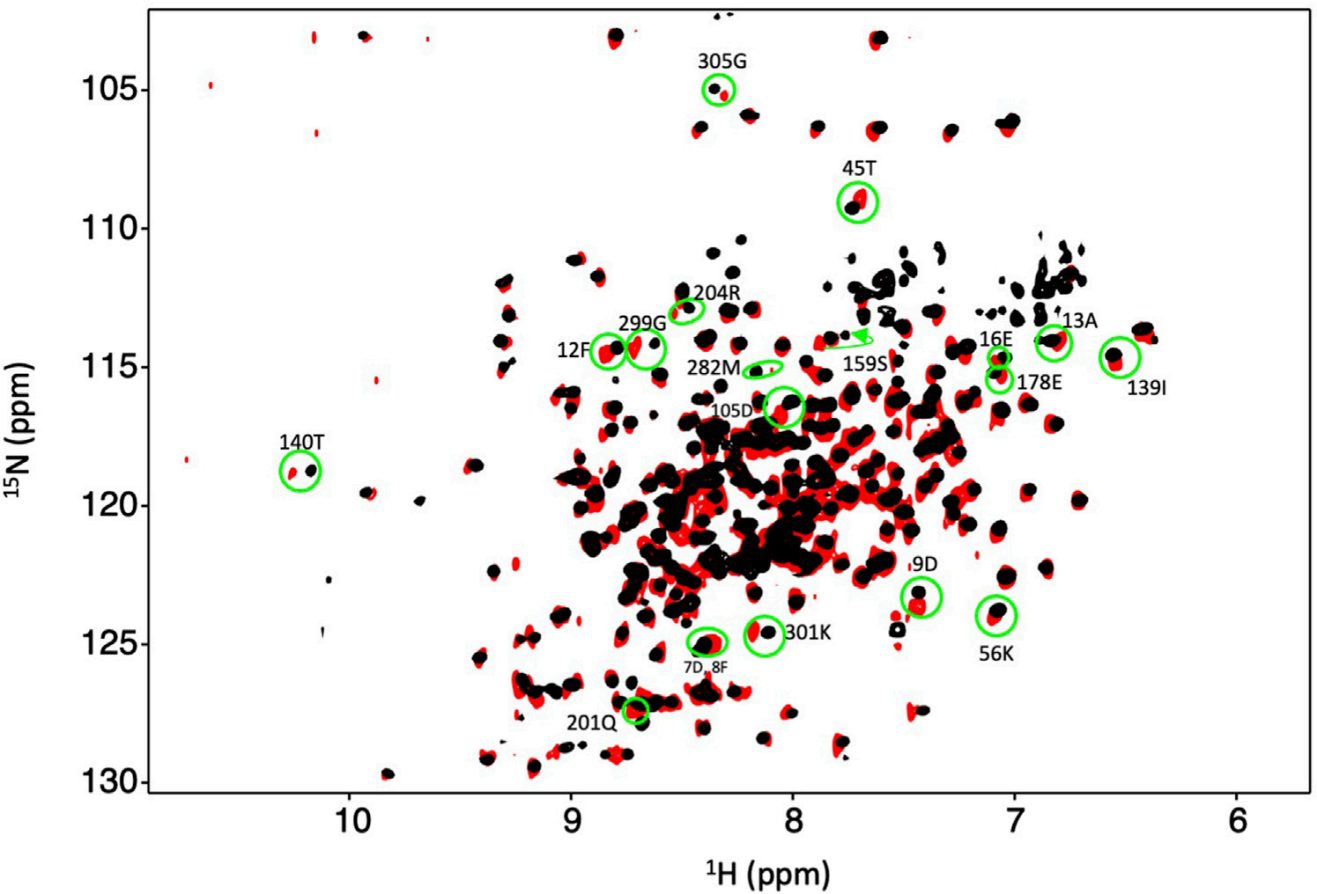
Dbh binds Mg^2+^ in the absence of DNA and nucleotide. Overlay of an ^15^N-HSQC of Dbh at 35°C with Mg^2+^ (black) and without Mg^2+^ (red). Shifted peaks are circled in green and the labeled with the residue number and the one letter code of the residue. Shifted peaks in the Dbh-Mg^2+^ spectra inform on local Mg^2+^ binding. Chemical shift changes were localized in three regions of the 3D structures (see Figure 7).

**FIGURE 7:**
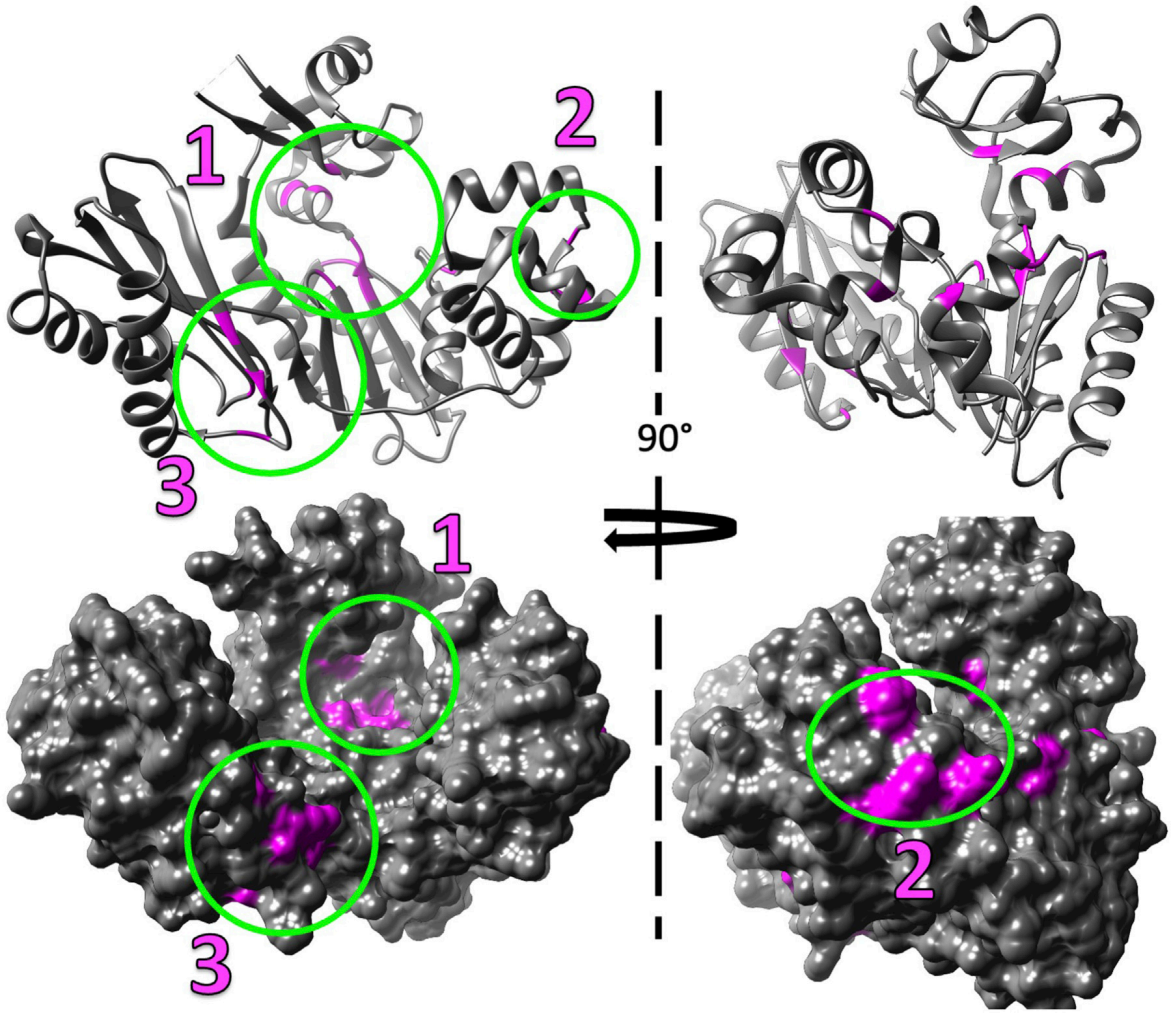
Mg^2+^ induced chemical shift changes map to three distinct locations when Dbh is in solution alone (without DNA and nucleotides). The shifted peaks from the overlayed ^15^N-HSQC of Dbh at 35°C with Mg^2+^ and without Mg^2+^ (Figure 6) were mapped on to the 3D structure of Dbh (PDB: 1K1S) shown as a ribbon diagram (top), and surface filled model (bottom). The shifted peaks were localized in three different locations: 1) at the active site in the palm/finger domain, 2) in the thumb domain, and 3) in the little finger domain.

Our studies show that Mg^2+^ interacts with Dbh in the absence of DNA. We analyzed ^15^N-HSQC spectra of Dbh in the presence and absence of Mg^2+^(Figure 6). When ligands, such as Mg^2+^, bind proteins they change the local chemical environment, resulting in chemical shifts changes for peaks of residues involved in ligand binding and for residues near the binding site or affected structurally by the binding event. Residues with shifted peaks in the Dbh/Mg^2+^ spectra inform us on local Mg^2+^ binding. We found shifted peaks clustered in three distinct locations: 1) at the active site in the palm/finger domains, 2) the thumb domain, and 3) the little finger domain (Figure 7). We found that ten of the residues that shift in the presence of Mg^2+^ are located in the active site. Seven of these are found in the palm domain and three in the finger domain (Table 2). Although they are in different domains, all residues are in proximity to the active site residues of this Y-family polymerase (Figure 7). Mg^2+^ also binds the thumb domain in the absence of DNA since the ^15^N-HSQC of Dbh in the presence of Mg^2+^ shows three peaks shift in the thumb domain (Table 2; Figure 7). In addition, we found that Mg^2+^ binds Dbh’s LF domain in the absence of DNA. When comparing the spectra with and without Mg^2+^ we observe that four peaks from the LF domain shift in the presence of Mg^2+^. All four residues are within 14 Å from each other in the three-dimensional structure (Table 2; Figure 7).

**TABLE 2:** List of residues peaks that disappeared in the presence of Mg^2+^or Mn^2+^ (Supplementary Figure S3). The domain, residue number, residue type, and binding site are given.

Domain	Residue #	Type of residue	Binding site	Mg^2+^	Mn^2+^
Palm	7	Asp	1	✓	✓
Palm	8	Phe	1	✓	✓
Palm	9	Asp	1	✓	✓
Palm	12	Phe	1		✓
Palm	13	Ala	1	✓	✓
Palm	16	Glu	1	✓	✓
Finger	25	Gly	1		✓
Finger	44	Ala	1		✓
Finger	45	Thr	1	✓	✓
Finger	58	Gly	1		✓
Palm	85	Ser	1		✓
Palm	89	Met	1		✓
Palm	103	Ser	1		✓
Palm	105	Asp	1	✓	✓
Palm	106	Glu	1		✓
Palm	108	Tyr	1		✓
Palm	117	Gly	1		✓
Palm	139	Ile	1	✓	✓
Palm	140	Thr	1	✓	✓
Palm	144	Gly	1		✓
Palm	145	Val	1		✓
Palm	156	Ala	1		✓
Palm	159	Ser	1	✓	
Palm	160	Lys	1		✓
Palm	162	Asn	1		✓
Palm	163	Gly	1		✓
Palm	165	Gly	1		✓
Thumb	177	Asn	2		✓
Thumb	178	Glu	2	✓	✓
Thumb	180	Asp	2		✓
Thumb	182	Asp	2		✓
Thumb	199	Gly	2		✓
Thumb	201	Gln	2	✓	✓
Thumb	204	Arg	2	✓	✓
LF	282	Met	3	✓	
LF	299	Gly	3	✓	✓
LF	301	Lys	3	✓	✓
LF	305	Gly	3	✓	✓

We confirmed ion binding sites by comparing ^15^N-HSQC spectra with and without Mn^2+^. Mn^2+^ binds proteins in a similar manner as Mg^2+^, however, Mn^2+^ is paramagnetic. This increases relaxation times, leading to the broadening and disappearance of peaks for residues near the binding site (16–25 Å) (Pintacuda et al., 2004). By overlaying the Mn^2+^ and apo spectra we should see the absence of peaks of residues near the metal binding sites (Supplementary Figure S5). The paramagnetic effect of Mn^2+^ is more far-reaching than chemical shift perturbations from Mg^2+^; consequently, the number of affected residues is increased in the Mn^2+^ samples compared to Mg^2+^ (Table 2). We observed the disappearance of 37 residues that correlate well with our predicted Mg^+2^ binding sites except for one (Gly117), which is not within any of our predicted binding sites (Supplementary Figure S6).

Although the high protein concentrations required for NMR studies complicate accurate calculations of dissociation constants (Jarmoskaite et al., 2020), we can compare relative affinities of Mg^2+^ binding sites using NMR. We measured chemical shift changes for the signals with the largest peak position difference upon Mg^2+^ binding in each site (Supplementary Figure S7). We find that all three binding sites have an affinity for Mg^2+^ in the low mM concentration range of the ion. The amount of endogenous Mg^2+^ in *S. acidocaldarius* has not been reported, but we do know that the concentration of Mg^2+^ in *E. coli* is between 30–100 mM (Milo and Phillips, 2015). We do not have experimental data on Mg^2+^ binding of the protein at physiological concentrations. However, we can say binding of Mg^2+^ to the protein at NMR concentrations is within the Mg^2+^concentration range of available ions in the cell. Thus, it is possible that Dbh binds Mg^2+^ under physiological conditions.

## 4 Discussion

Most of the information on Y-family polymerase dynamics has originated from a related polymerase known as Dpo4, which has been studied to a greater extent. Dpo4 has over 90 crystal structures, and multiple MD simulations (Wang et al., 2005; Chu et al., 2014; Liyanage et al., 2017; Chu et al., 2021) compared to Dbh with only seven crystal structures and no simulations. Dpo4 has been studied in multiple states which has allowed researchers to implement MD simulations to understand the motional properties of the enzyme. MD simulations have shown that Dpo4 maintains conformational flexibility even after binding DNA (Chu et al., 2021). Some of these dynamics may translate to Dbh but since Dbh and Dpo4 are only 54% identical (Boudsocq et al., 2004) it may be that Dbh has unique dynamic features. Expecting distinct flexibility is consistent with the rate of reaction where Dbh is slower (*k*_*p*_ = 0.64 s-1 at 25°C) (Cramer and Restle, 2005) than Dpo4 (*k_p_* = 6.4 s-1 at 26°C) (Fiala and Suo, 2004), and different fidelity since Dbh has been shown to synthesize with higher fidelity than Dpo4 (Boudsocq et al., 2004). The MD simulation studies conducted with Dpo4 in complex with DNA do not address the issue of temperature which is relevant to both proteins.

### 4.1 Effect of temperature on Dbh structure

NMR ^15^N-HSQC chemical shifts reveal that Dbh is thermally stable; however, tertiary structure denatures at and below 25°C. The protein maintains secondary structure down to 2°C as determined by CD (Figure 4) and remains compact and monomeric (DLS). Together these data show that Dbh retains tertiary structure between 35°C–65°C, and transitions to a molten globule at and below 25°C. Cold denaturation is unusual for a large protein and could be related to the requirement for this protein to function at very high temperatures.

Although many proteins crystallize well in the cold, it is notable that Dbh crystals formed best at room temperature (Silvian et al., 2001; Wilson and Pata, 2008; Wilson et al., 2013). In addition, the loss of tertiary structure at 25°C correlates well with the loss of catalytic efficiency observed in a primer extension study at 22°C compared to higher temperatures. Potapova, et al., reported a 40-fold change in reaction rate going from 65°C to 22°C (Potapova et al., 2002). However, kinetic theory predicts a two-fold change in rate for every 10°–if the transition state remains the same. If the mechanism were maintained across the temperature range, an approximate change in temperature of ∼40°C should have resulted in an approximate 16-fold change in rate of synthesis, much smaller than the 40-fold rate change that Potapova, et al. reported. It is likely that structural changes resulting from cold denaturation contribute to an additional 2.5-fold diminishment of catalytic efficiency measured at 22°C.

### 4.2 Backbone dynamics

Using PFs we determined the regions/domains of the protein that are resistant to exchange and compared these regions of rigidity at a temperature close to the cold denaturation transition (35°C) and one closer to the optimal functional temperature (50°C). There are two mechanisms that could affect rates of amide exchange: changes in local unfolding kinetics or a structural change whereby an amide becomes more or less occluded from solvent. For proteins where the structure does not change significantly, differences in the rates of amide hydrogen exchange can reflect the influence of the temperature on the local unfolding of that amide hydrogen bond.

As previously mentioned Dbh functions in extremely hot environments (∼75°C), therefore the dynamics we observed at temperatures close to Dbh’s natural environment should reflect the natural state of the Dbh polymerase. In general, our protection factor data show that Dbh protein is considerably more dynamic at 50°C than at 35°C. We mapped PFs at 35°C and 50°C on the crystal structure of Dbh (Figure 5) and a clear pattern of dynamics and stability emerged. PFs show that the palm domain and the little finger domain are the most stable regions of Dbh at both temperatures. In contrast, the dynamics of the thumb and finger domain are significantly different between 35°C and 50°C. At 35°C we observe nine residues protected in the finger domain and six residues protected in the thumb domain. This protection is lost at 50°C indicating that these domains become more flexible as the temperature is increased toward the physiological temperature of *S. acidocaldarius*.

The palm domain is the most stable domain in Dbh (Figure 5). The LF domain is the second most stable part of Dbh, followed by the fingers domain. Compared to the other domains the thumb domain seems to be most dynamic regardless of temperature. This is revealed by a lack of protected amides. Nevertheless, like the fingers domain, the thumb domain shows the same trend with lower PFs at 50°C than 35°C. At 50°C there are no protected residues in the Dbh thumb domain. TC data show that residue 176 is sensitive to temperature increase (TC value = −6.61), indicating unfolding with temperature. Together the protection factor data and TC calculations show that the thumb domain is the most flexible domain in the protein and that this flexibility increases with temperature.

TC values for proteins have been shown to be sensitive to temperature dependent global and local unfolding (Ohnishi and Urry, 1969; Andersen et al., 1997; Baxter and Williamson, 1997). Historically TC data for protein is analyzed at the individual amide level. This correlates with our PF data that show that a small region of the palm domain becomes slightly more flexible when going from 35°C to 50°C (Figure 5). Together these two methods allow us to conclude that although the palm domain is the most protected domain at both temperatures, helix E (77-94) and helix F (121–138) of the palm domain do adopt flexibility as temperature increases.

Most recently, analysis of TC data has been performed in group averages of helix secondary structures (Doyle et al., 2016). It has been shown that this type of analysis is a rich source of information on temperature dependent global stability (Doyle et al., 2016). We averaged our TC data by secondary structures, and by domain (Table 1). When averaging by secondary structure (*i.e.,* strand and helix) only limited structural information is obtained. In contrast, averaging the entire domains shows a clear trend that correlates with our hydrogen exchange PF data. We see the most dynamic domain the thumb domain has the most negative TC average of −3.28 ppb/K, followed by the second most dynamic finger domain with a TC domain average of −3.19 ppb/K. For the more stable domains the TC averages are close, but do not correlate with our PF data. TC average for the palm domain was −2.77 ppb/K and −2.69 for the LF domain.

Other groups have correlated TC and HX to obtain structural information on proteins (Andersen et al., 1997; Baxter and Williamson, 1997). It has been shown that protected amides (slow exchangers) average a less negative TC average compared to lower protected amides (fast exchangers) who have a more negative TC average (Andersen et al., 1997). Our TC average analysis only included amides in defined secondary structure (strands and helix). The analysis excluded amides in unstructured regions and amides that did not hydrogen bond and were exposed to water. This was done to avoid artificially decreasing our average TC by including naturally fast exchanging amides.

### 4.3 Metal ion binding

Chemical shift changes of ^1^H-^15^N HSQC reveal three ion binding sites of Mg^2+^ and Mn^2+^, even in the absence of bound DNA. The binding regions include two that have been reported previously in crystal structures of other Y-family polymerases in the presence of DNA (Irimia et al., 2010; Pata, 2010) and a novel binding region on the LF domain of Dbh. There is currently an active debate on whether the DNA synthesis mechanism requires two or three ions for synthesis. The third ion has been suggested to be involved in the active site interacting with the incoming nucleotide (Yang et al., 2016). Our experiments did not involve DNA or free-floating nucleotide. However, a third binding site is apparent even without DNA bound.

Twenty-three residues that disappeared in the presence of Mn^2+^ are near the palm/finger Mg^2+^ binding site, three are found near the LF Mg^2+^ binding site, and ten were in the thumb Mg^2+^ binding site (Table 2; Figure 7). Notably, all residues that shift in presence of Mg^2+^ spectra also disappear in the Mn^2+^ spectra (Table 2) except for residue Met282. It is possible that Met282 chemical shift changes with Mg^2+^ are a result of long-range conformational change and this residue may not be involved directly in ion binding. Regardless, the Mn^2+^ data serves as a secondary verification of our proposed ion-binding sites.

Notably, apo crystal structures of Dbh do not show binding of metal ions (PDB: 1IM4 (Zhou et al., 2001), 1K1Q (Silvian et al., 2001), 1K1S (Silvian et al., 2001)). In contrast, Dbh in complex with DNA has been shown to bind one Ca^2+^ (Wilson and Pata, 2008). The chemical shift changes we observed indicate that Mg^2+^ binds Dbh in the active site in the absence of DNA in solution; however, these ions bind weakly since they do not co-crystallize with the protein. The active site of Y-family polymerases consist of the palm domain surrounded by the finger domain and to a lesser extent the thumb domain (Wilson and Pata, 2008; Irimia et al., 2010; Pata, 2010). One Ca^2+^ binds in the active site in Dbh/DNA structures (Wilson and Pata, 2008). This Ca^2+^ ion is chelated by Asp7, Phe8, and Asp105. Our solution-state NMR data show that Mg^2+^ interacts with these same residues in Dbh even without DNA bound. Furthermore, these chelating residues are surrounded by residues Asp9, Ile139, and Thr140; we find that these also experience chemical shift changes in the presence of Mg^2+^. In addition, our data shows that Mg^2+^ interacts with three residues that are near the active site but shifted towards the finger domain, these include Ala13, Glu16, and Thr45. It may be that Mg^2+^ is positioned more towards the finger domain when Dbh is in solution and not in the presence of DNA or that these residues are structurally adjusted when the ion binds.

As mentioned earlier other Y-family such as Dpo4 polymerase have been shown to crystalize with two Mg^2+^ ions near this active site. This interaction can be attributed to a second Mg^2+^ which is almost always shown interacting with DNA and the protein. Published studies (Irimia et al., 2010) show Dpo4 chelating Mg^2+^ with residues 181–183 (182–184 Dbh). Our work suggests a slightly different binding site near residues 178, 201, and 204 in the absence of DNA. Two residues in this vicinity are positively charged Arg204 and Lys202. The peak corresponding to Arg204 is shifted in the presence of Mg^2+^, but the peak corresponding to Lys202 is found in a crowded region of the spectra which could not be accurately measured. Both charged residues have potential salt bridge partners, however, one appears to be too far for the average salt bridge distance of 4 Å (204Arg-205Asp (5.2Å) and 202Lys-180Asp (2.7 Å)). If they are indeed forming salt bridges the introduction of Mg^2+^ in this region could interrupt the salt-bridge partners which could lead to peak shifts caused by the repulsion forces of two like charges.

Our results show that the Dbh LF has a Mg^2+^ binding site. Dpo4 has been shown to bind a Mg^2+^ ion in the LF domain near Asp294 (PDB 2XCA) (Irimia et al., 2010). However, we found the Dbh binding site to be 23.5 Å away from the reported binding site of Dpo4. Dbh and Dpo4 sequences have the lowest sequence similarity between their LF domains with only 42.1% similarity. These amino acid changes could result in a movement of the ion binding site. Furthermore, a sequence alignment across 34 Y-family homologs showed that two of the shifted residues (Gly299, Lys301), are commonly mutated in other Y-family polymerases (Wu et al., 2017). We suspect that the distinct sequence identity of the LF domain may create a Mg^2+^ binding region unique to Dbh. However, another explanation for the lack of Mg2+ binding in the LF in other crystal structures might be caused by low occupancy Mg2+. It is difficult to visualize using crystallography if the ion has lower than 30% occupancy due to the low electron number of Mg2+ (Yang et al., 2016).

## 5 Conclusion

Here we present preliminary studies on the bypass polymerase Dbh in solution. We found several novel features of this protein by studying the protein in solution. Protection factors from NMR hydrogen exchange (HX) experiments of backbone amides at 35°C and 50°C reveal changes in dynamics that could drive the functionality of Dbh. We find that palm and LF domains remain stable at 35°C and 50°C, with a minor increase in dynamics at 50°C. The thumb and finger domains are revealed to be the most flexible domains on Dbh, with the dynamics significantly increased at 50°C. Temperature coefficient (TC) calculations correlate with dynamics observed in the protection factor analysis. The palm and LF domains are the most thermally stable. Maintaining the palm structure at high temperatures is likely a requirement to ensure function. NMR results show that the LF is folded at high temperatures with PFs that reveal it to be more stable than the fingers and thumb domains. Interestingly, the LF of Dpo4 was found to be the most stable domain in thermal denaturation studies (Sherrer et al., 2012). A rationale for the thermal stability of the LF domain is not clear at this time since the function of this domain remains to be defined.

Dbh shows a cold denaturation transition above zero degrees. It is unusual to observe cold denaturation significantly above freezing and is likely to be related to the evolution of this enzyme to function at high temperatures. We also find evidence for three divalent ion binding sites, even in the absence of DNA/nucleotides. To our knowledge, this is the first study to report Dbh directly interacting with Mg^2+^ and Mn^2+^ without DNA. Consequently, it appears that three metals may be co-localized to the protein making them readily available for DNA binding.

## Data Availability

The original contributions presented in the study are included in the article/Supplementary Material, further inquiries can be directed to the corresponding author.

## Abbreviations

DinB homolog polymerase (Dbh); HX, hydrogen exchange rate; k_ex_, amide measured exchange rate; k_rc_, exchange rate of amide in a random coil; k_p_, polymerization rate; LF, little finger domain; MD, molecular dynamics; PF, protection factor; TC, temperature coefficient.
