# Supplementary material for: Dynamics and thermal stability of the bypass polymerase, DinB homolog (Dbh)
Source: Front Mol Biosci. 2024 Apr 30;11:1364068. doi: 10.3389/fmolb.2024.1364068 (PMC11091320; doi:10.3389/fmolb.2024.1364068)
Supplement: Supplementary file 1 [file DataSheet1.PDF]

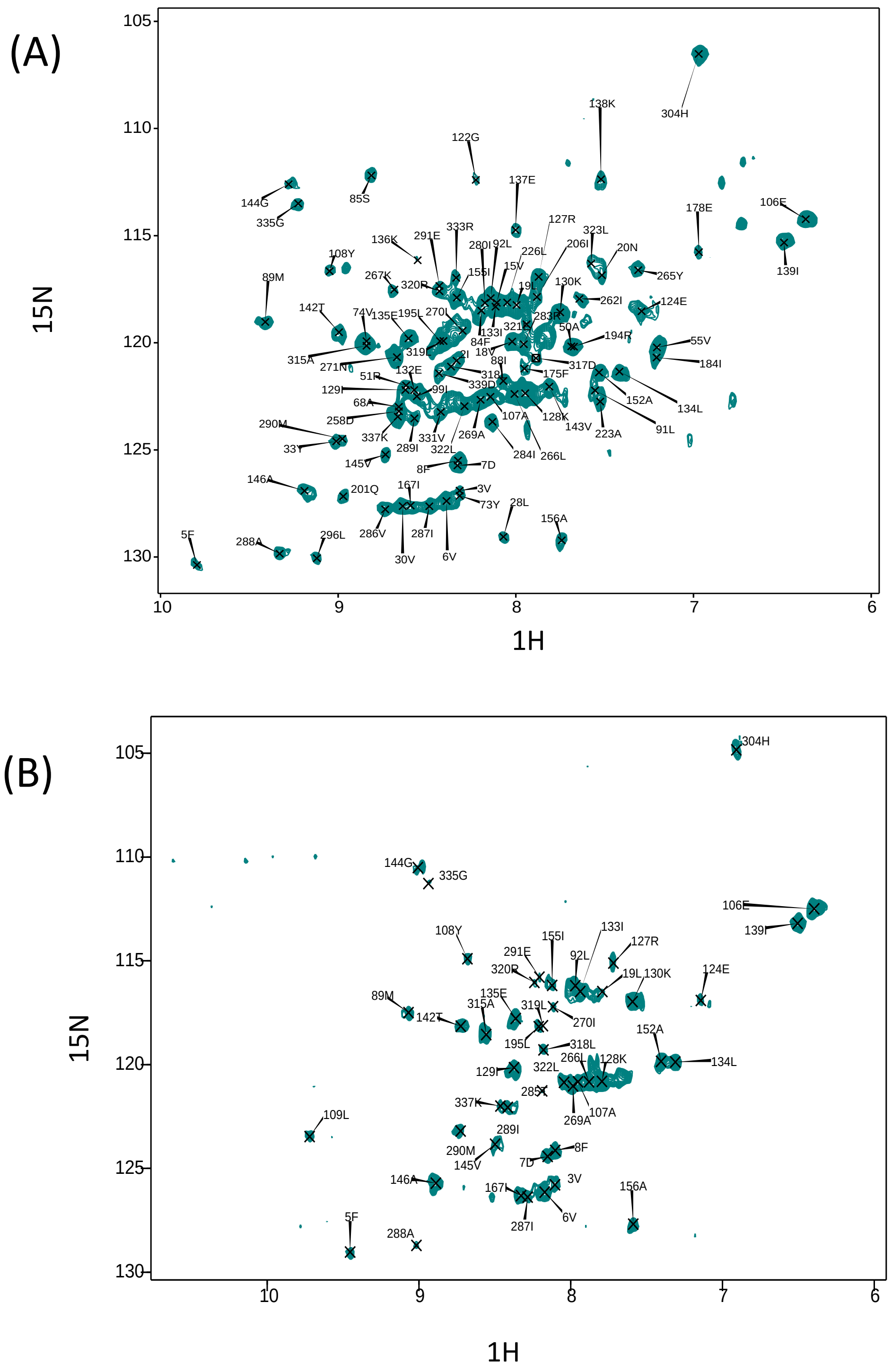

Figure S1: Peak assignments of the first time point after initiation of exchange with D_2O for Dbh at (A) 35 °C (16 minutes) and (B) 50 °C (15 minutes), ^{15}N-HSQC.

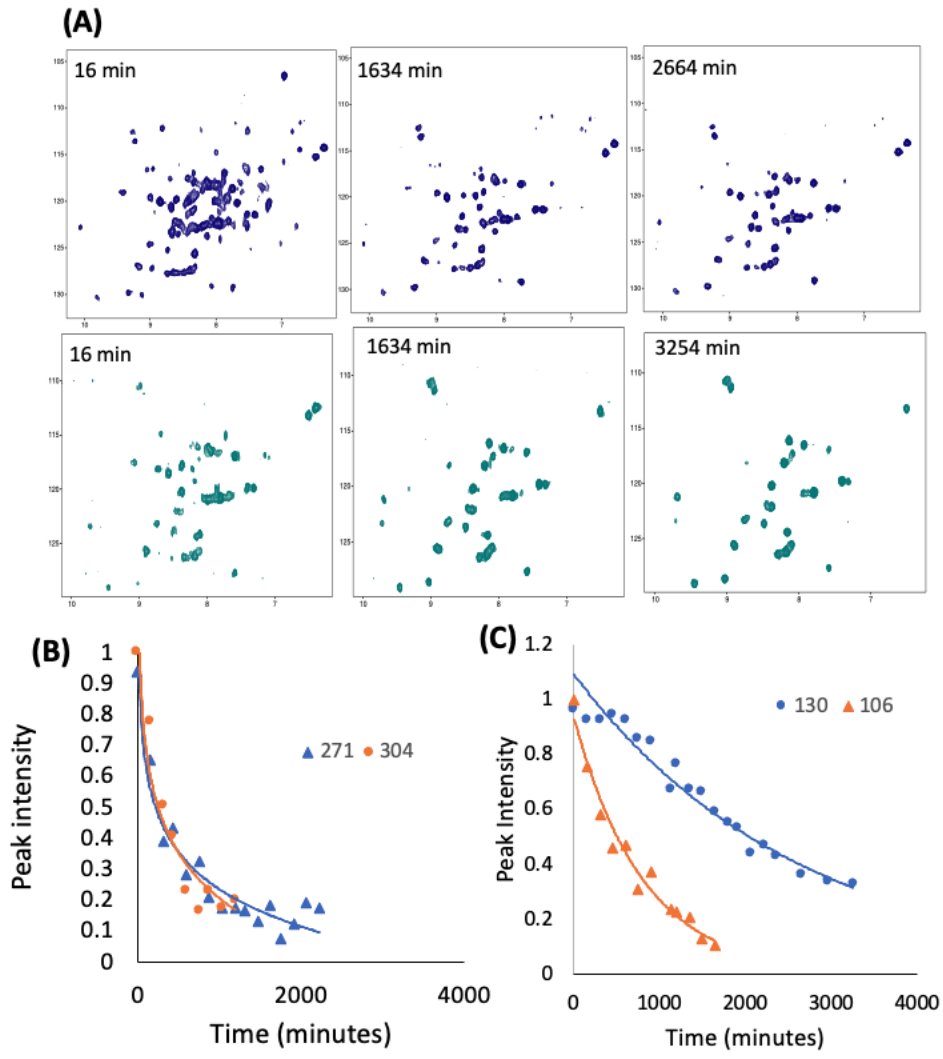

Figure S2: (A) ^{15}N-HSQC spectra of Dbh at 35 °C (blue, top) and 50 °C (teal, bottom) at different time points after being transferred into D_2O. Peaks were integrated at each time point over a period of two weeks and plotted as a function of time. Hydrogen exchange rates (k_{ex}) were calculated from a fit to a single exponential. Representative fits: **(B)** 35 °C: 271Asn (blue, ▲) and 304His (orange, ●) hydrogen signals as a function of time. **(C)** 50 °C: 130Lys (blue, ●) and 106Glu (orange, ▲) hydrogen signals as a function of time.

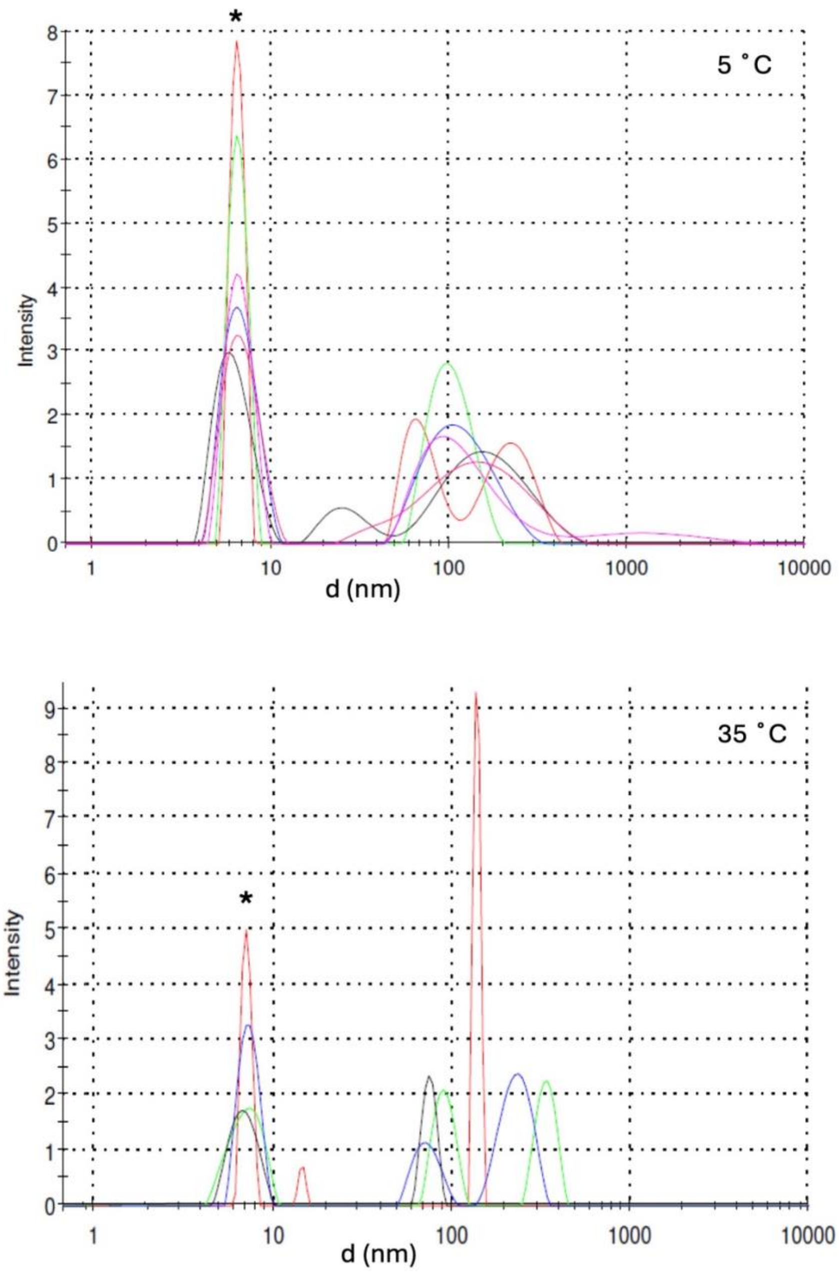

Figure S3: Dynamic Light Scattering (DLS) of a diluted, unfiltered Dbh NMR samples, particle diameter (d) at 5°C (top panel) and 35°C (bottom panel), by intensity. The size of Dbh monomer (*) is between 6-7nm diameter. There is slight decrease in diameter at 5°C for the monomer. These NMR samples contained a miniscule amount of high-order aggregates. Note that the intensity scale for DLS is not linear with particle diameter since large particles scatter light exponentially more efficiently. Intensity scales with a particle diameter power of 6 (d^6). Consequently, if a specific concentration of monomers has a 7 nm diameter (1X, d^6 = 117,649), the same concentration of monomers aggregated to form dimers would produce a scattering signal that is 32X the 7 nm intensity (14 nm is a d^6 = 7,529,536 = 64X compared to 7 nm for the particles, two monomers per particle). Similarly, a 70 nm particle would have an intensity of 1,000,000X and 140 nm would be 64,000,00X compared to the 7 nm intensity. Although signals for large species appear comparable to the 6-7 nm peak intensities, they represent an extremely small amount of protein that would not be visible in the NMR spectrum.

(A)

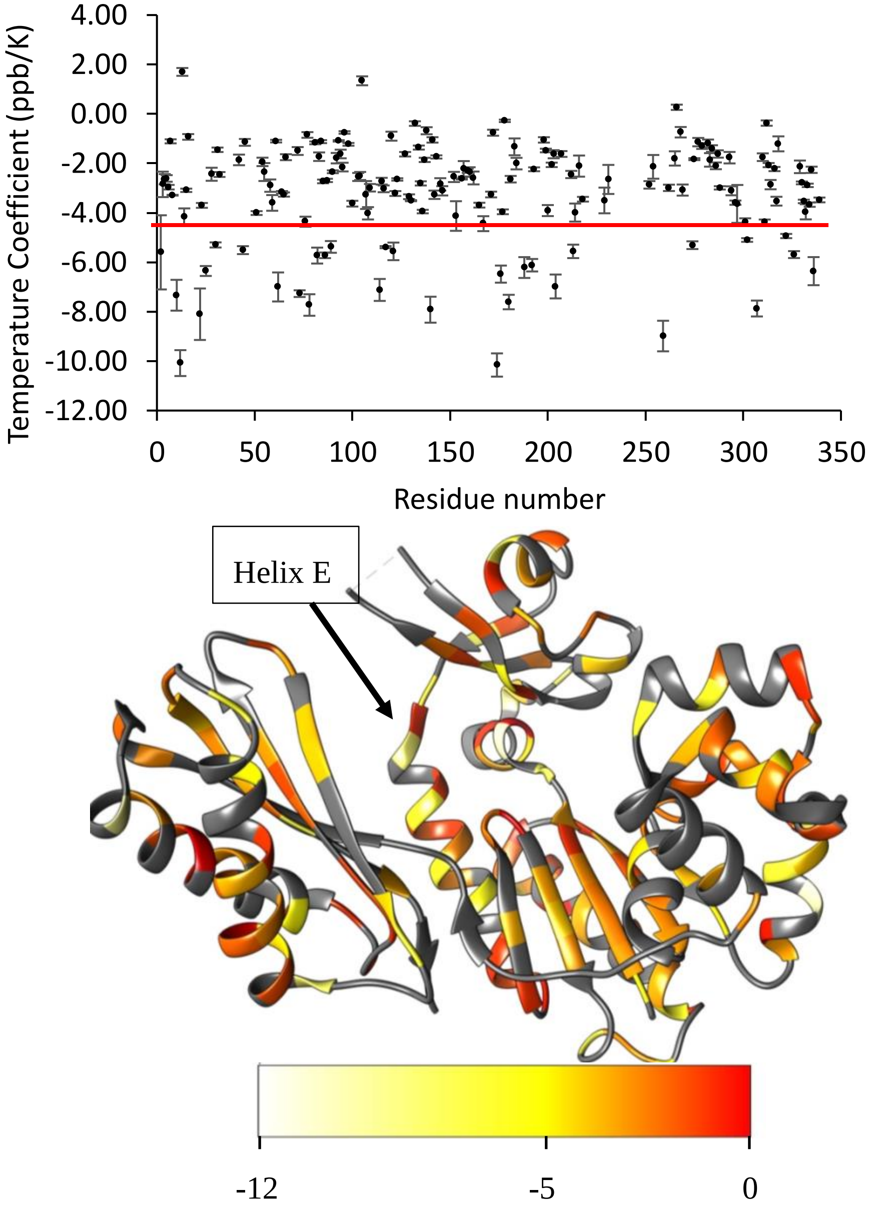

(B)

Figure S4: (A) Temperature coefficient values for amides in Dbh. The red line is a threshold (-4.6 ppb/K) for amides hydrogen bonding with water. (B) TC values mapped on to the 3D structure of Dbh (PDB: 1K1S). Most residues with values more negative than -4.6 ppb/K belong to amides that are exposed to water or experience unfolding as a function of temperature. For example, helix E (77-94) of the palm domain has four residues which are involved in intramolecular hydrogen bonding but have TC values more negative than -4.6 ppb/K threshold. This indicates this region is susceptible to unfolded as temperature increase. This flexibility in helix E is also reflected in the protection factor data.

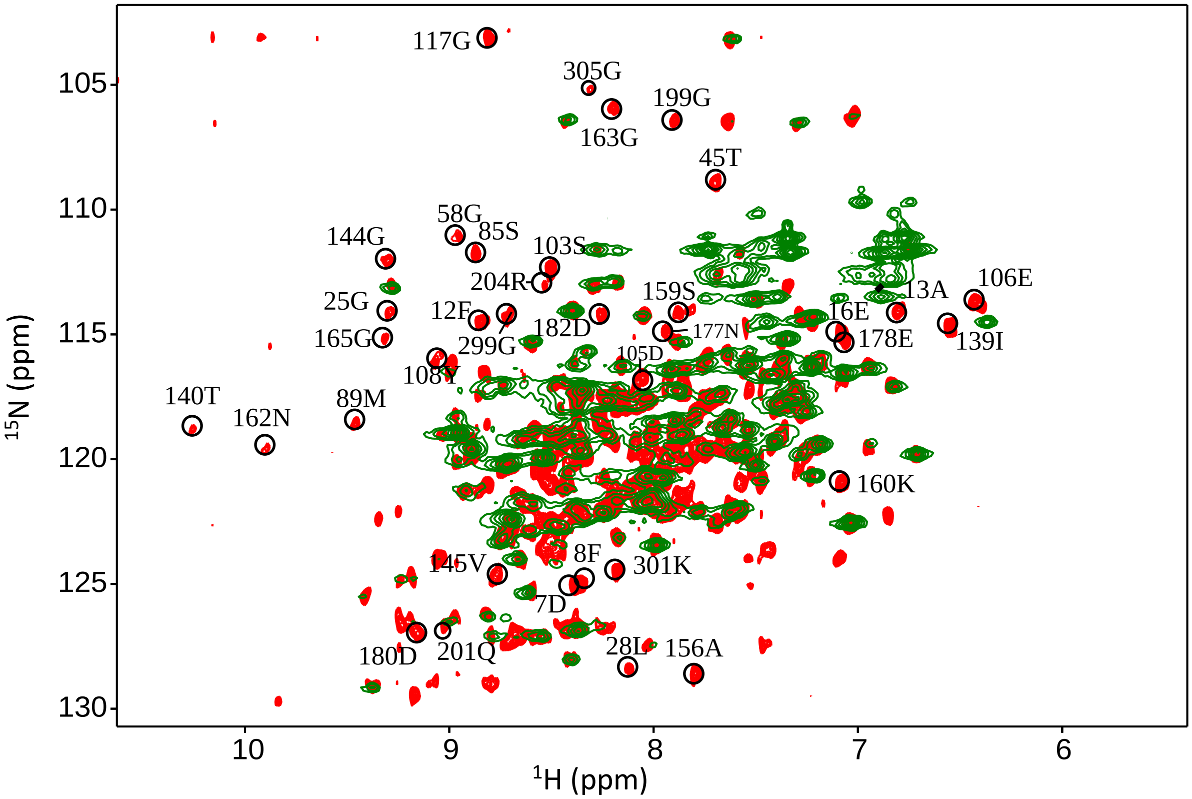

Figure S5: Verification of Mg^{2+} binding sites with Mn^{2+}. Mn^{2+} binds proteins similar Mg^{2+}; however, it has paramagnetic properties that increase relaxation times leading to broadening of peaks for residues near the ion binding site. We observed 37 ^{15}N-HSQC peaks disappear in the presence of Mn^{2+}. These are in proximity to a Mg^{2+} binding site predicted by chemical shift mapping. It is important to note that all residues that shifted in the Mg^{2+} spectra also disappeared in the Mn^{2+} spectra except for residue 282Met.

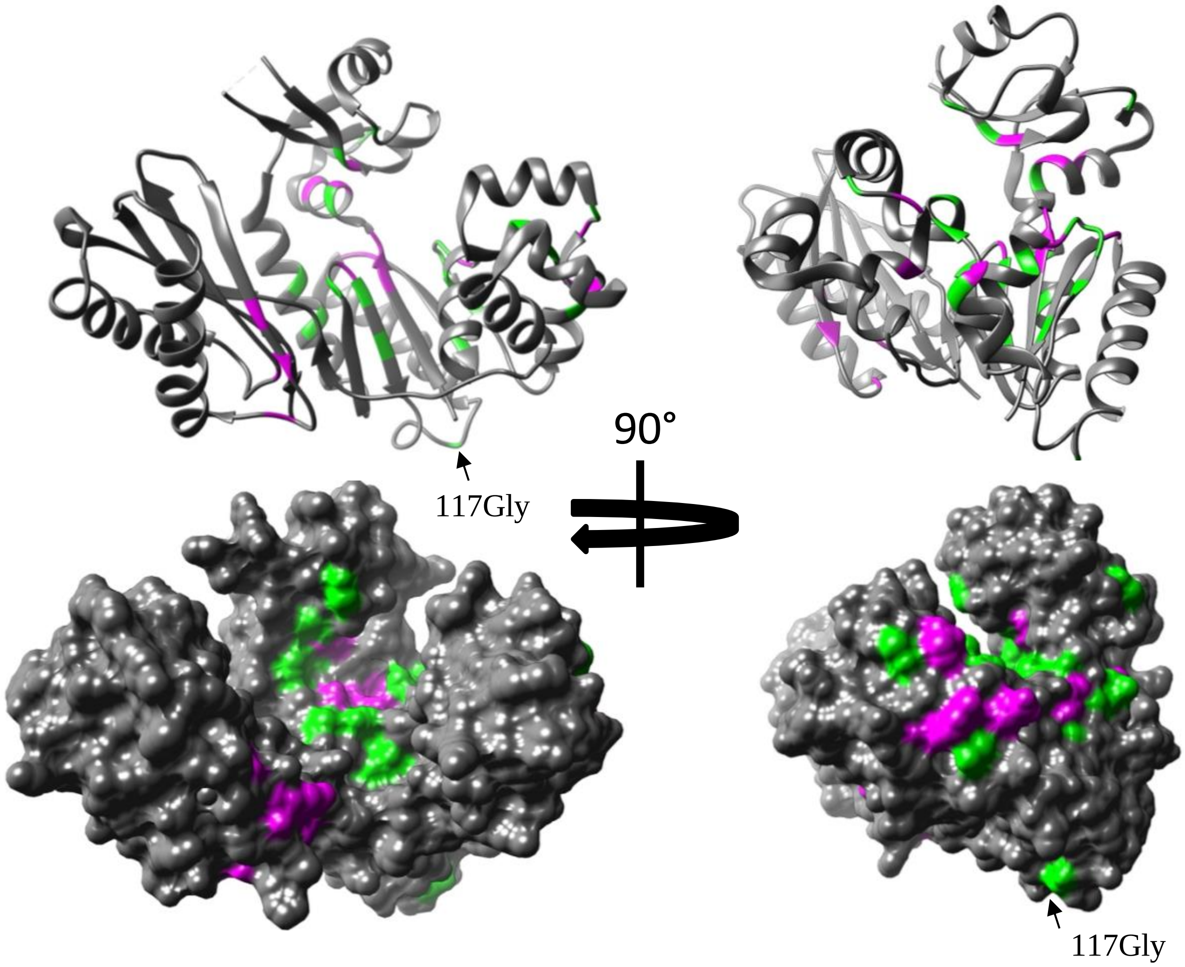

Figure S6: Mg^{2+} and Mn^{2+} binding sites. The residues that are broadened beyond detection within the Dbh/Mn^{2+} ^{15}N-HSQC are mapped onto the structure (PDB: 1K1S). We find that 36 of 37 residues are localized in one of the three predicted Mg^{2+} binding cites. Mn^{2+} paramagnetic properties extend further than the effect of Mg^{2+} binding. The only residue that did not localized near the binding site was Gly117 which is found 23.0 Å to the nearest predicted binding site (palm/finger domain).

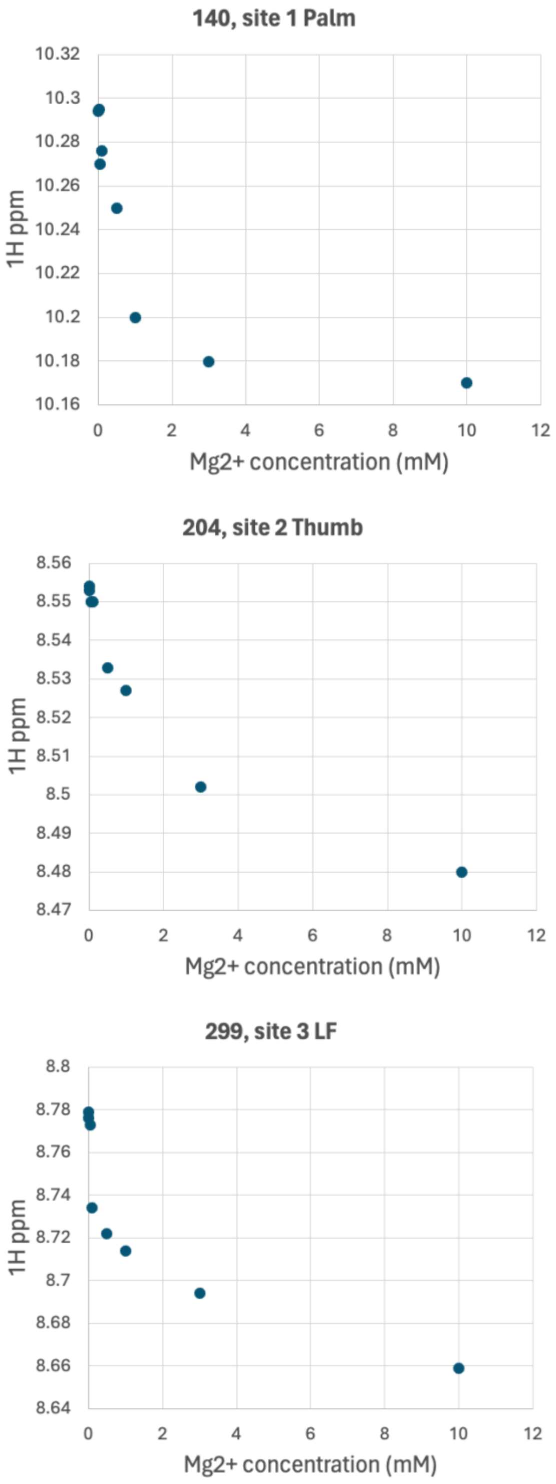

Figure S7: Mg^{2+} titration. Chemical shift changes of ^{1}H-^{15}N HSQC peaks for residues 140, 204 and 299 with increasing Mg^{2+} concentrations: 0, 0.01, 0.05, 0.1. 0.5, 1, 3, 10 mM. The Dbh concentration was 0.3 mM.

Table S1. Measured exchange rates (k_{ex}) and Protection Factors (PF) at 35 °C and 50°C for all detected amides. Also includes Temperature Coefficient (TC). Cells with 'stable' showed no signs of decay overall the course of the experiment. Cells denoted with these symbols (*, †, ‡) exchange too fast to fit; these completely exchanged after 10 min (*), 16 min (†), or 310 min (‡).

Residue #	k_{ex} ± error at 35 °C	PF at 35 °C	k_{ex} ± error at 50 °C	PF at 50 °C	TC ± S.E of the slope
2	6.4E-04 ± 9.7E-05	6.65E+06			-5.60 ± 1.50
3			stable	stable	-2.85 ± 0.51
4					-2.64 ± 0.21
5	stable	stable	stable	stable	-2.61 ± 0.13
6	stable	stable	stable	stable	-2.96 ± 0.09
7	5.1E-05 ± 1.4E-05	6.76E+07	stable	stable	-1.11 ± 0.08
8	8.4E-05 ± 1.6E-05	6.19E+07	1.65E-03 ± 2.90E-04	1.37E+07	-3.29 ± 0.01
9					Non-linear
10					-7.33 ± 0.62
12					-10.08 ± 0.52
13					1.70 ± 0.16
14					-4.15 ± 0.33
15	2.2E-04 ± 2.6E-05	3.20E+07			-3.09 ± 0.07
16					-0.93 ± 0.11
18	9.0E-04 ± 6.2E-05	6.09E+06			
19	2.3E-04 ± 7.9E-05	6.43E+06	1.38E-04 ± 5.03E-05	3.95E+07	

20	*	*			
22					-8.10 ± 1.04
23					-3.70 ± 0.10
24					
25					-6.35 ± 0.20
28	3.5E-04 ± 5.8E-05	6.95E+06			-2.44 ± 0.26
29					
30	3.7E-04 ± 3.9E-05	9.98E+06			-5.30 ± 0.10
31					-1.45 ± 0.09
32					-2.45 ± 0.10
33			1.5E-04 ± 3.7E-05	2.82E+07	
42					-1.86 ± 0.21
44					-5.51 ± 0.16
45					-1.15 ± 0.14
50	1.3E-03 ± 8.7E-05	2.17E+07			
51					-4.01 ± 0.08
53					Non-linear
54					-1.94 ± 0.17
55	7.2E-03 ± 2.3E-03	3.46E+05			-2.35 ± 0.36
56					Non-linear

58					-2.90 ± 0.25
59					-3.60 ± 0.32
61					-1.10 ± 0.05
62					-7.00 ± 0.59
63					Non-linear
64					-3.17 ± 0.05
65					-3.26 ± 0.09
66					-1.75 ± 0.12
68	*	*			
72					-1.50 ± 0.16
73					-7.27 ± 0.14
74	1.1E-04 ± 1.2E-05	5.09E+07			
76					-4.36 ± 0.20
77					-0.85 ± 0.11
78					-7.73 ± 0.44
80					Non-linear
81					-1.17 ± 0.06
82					-5.73 ± 0.32
83					-1.73 ± 0.17
84	2.7E-04 ± 8.9E-05	1.73E+07			-1.12 ± 0.06

85	2.4E-03 ± 5.6E-04	1.29E+07			-2.73 ± 0.07
86					-5.72 ± 0.11
87					-2.69 ± 0.07
88	4.5E-05 ± 2.4E-05	3.40E+07			
89	1.9E-04 ± 3.7E-05	4.11E+07	†	†	-5.38 ± 0.24
90					-2.34 ± 0.06
91	8.8E-04 ± 1.8E-04	8.20E+06			
92	2.1E-04 ± 3.1E-05	1.40E+07	2.64E-03 ± 4.36E-04	3.88E+06	-1.79 ± 0.21
93					-1.09 ± 0.03
94					-1.62 ± 0.17
95					-2.15 ± 0.18
96					-0.75 ± 0.05
98					-1.23 ± 0.06
99	*	*			
100					-3.63 ± 0.12
103					-2.55 ± 0.16
104					-2.51 ± 0.16
105					1.34 ± 0.18
106	1.4E-04 ± 1.2E-05	4.49E+07	1.29E-03 ± 8.75E-05	2.43E+07	Non-linear
107					-3.28 ± 0.57

108	stable	stable	1.20E-03 ± 4.52E-04	1.24E+07	-4.02 ± 0.25
109	stable	stable	stable	stable	-2.99 ± 0.15
114					-7.12 ± 0.44
115					-2.72 ± 0.14
116					-3.03 ± 0.14
117					-5.39 ± 0.04
120					-0.90 ± 0.18
121					-5.56 ± 0.36
122	*	*			-3.22 ± 0.10
123					-2.65 ± 0.05
124	1.9E-04 ± 3.7E-05	1.31E+07	1.59E-03 ± 6.89E-04	2.75E+06	
127	1.6E-03 ± 1.8E-04	1.82E+07	*	*	-1.63 ± 0.09
128	stable	stable	stable	stable	
129	stable	stable	stable	stable	-3.35 ± 0.10
130	stable	stable	3.55E-04 ± 2.08E-05	2.69E+08	-3.51 ± 0.02
132					-0.39 ± 0.09
133	9.7E-05 ± 1.9E-05	1.81E+07	stable	stable	
134	stable	stable	2.33E-04 ± 3.64E-05	1.40E+08	-1.36 ± 0.08
135	4.2E-04 ± 2.6E-05	1.94E+07	‡	‡	-2.81 ± 0.08
136	*	*			-3.95 ± 0.07

137	*	*			-1.86 ± 0.09
138	2.0E-04 ± 1.1E-04	1.64E+07			-0.68 ± 0.14
139	stable	stable	1.28E-04 ± 2.75E-05	9.29E+07	Non-linear
140					-7.92 ± 0.53
141					-1.05 ± 0.11
142	stable	stable	2.68E-04 ± 3.81E-05	1.69E+08	-3.27 ± 0.17
143	6.8E-05 ± 2.8E-05	3.03E+07			-1.74 ± 0.06
144	stable	stable	stable	stable	Non-linear
145	stable	stable	stable	stable	-2.85 ± 0.25
146	Stable	Stable	stable	stable	-3.11 ± 0.19
152	stable	stable	stable	stable	-2.55 ± 0.21
153					-4.13 ± 0.60
155	stable	stable	stable	stable	
156	stable	stable	stable	stable	-2.63 ± 0.02
157					-2.22 ± 0.31
159					-2.30 ± 0.08
160					-2.35 ± 0.19
161					0.00 ± 0.00
162					-2.59 ± 0.26
163					Non-linear

165					-3.69 ± 0.10
167	3.3E-04 ± 3.1E-05	1.18E+07	stable	stable	-4.44 ± 0.30
171					-3.26 ± 0.12
172					-0.76 ± 0.12
173					0.00 ± 0.00
174					-10.16 ± 0.47
175	‡	‡			
176					-6.48 ± 0.35
177					-3.96 ± 0.10
178	†	†			-0.28 ± 0.05
180					-7.61 ± 0.29
181					-2.64 ± 0.13
182					Non-linear
183					-1.33 ± 0.34
184	†	†			-2.01 ± 0.23
188					-6.21 ± 0.42
192					-6.12 ± 0.25
193					-2.24 ± 0.08
194	1.4E-03 ± 9.7E-05	5.53E+07			
198					-1.05 ± 0.11

199					-1.48 ± 0.07
200					-3.91 ± 0.22
201	4.6E-04 ± 3.7E-05	4.89E+07			
202					-2.06 ± 0.09
203					-1.63 ± 0.16
204					-6.98 ± 0.48
206	†	†			
207					-1.62 ± 0.12
212					-2.45 ± 0.15
213					-5.56 ± 0.27
214					-3.99 ± 0.37
216					-2.11 ± 0.43
218					-3.45 ± 0.10
223	*	*			Non-linear
229					-3.50 ± 0.52
231					-2.65 ± 0.59
252					-2.87 ± 0.15
254					-2.14 ± 0.48
258	stable	stable			
259					-8.99 ± 0.62

262	6.4E-04 ± 1.1E-04	3.15E+06			-2.99 ± 0.13
265	‡	‡			-1.80 ± 0.29
266	stable	stable	1.56E-04 ± 3.46E-05	8.90E+07	0.27 ± 0.11
267	‡	‡			
268					-0.74 ± 0.21
269					-3.08 ± 0.22
270	stable	stable	stable	stable	
271	1.4E-03 ± 1.7E-04	2.82E+07			
274					-5.31 ± 0.16
275					-1.83 ± 0.04
277					-1.14 ± 0.17
278					
279					-1.30 ± 0.12
280	1.6E-04 ± 2.8E-05	2.61E+07			
282					-1.19 ± 0.15
283	‡	‡			-1.86 ± 0.27
284	stable	stable			-1.40 ± 0.13
285			1.43E-04 ± 1.10E-04	4.75E+07	
286	stable	stable			-2.10 ± 0.14
287			stable	stable	-1.62 ± 0.15

288	stable	stable	stable	stable	-2.99 ± 0.07
289	stable	stable	stable	stable	
290	stable	stable	stable	stable	
291	3.6E-04 ± 3.3E-05	3.26E+07	*	*	
293					-1.77 ± 0.23
294					-3.11 ± 0.18
296	1.1E-03 ± 2.8E-04	1.50E+06			-3.59 ± 0.09
301					-4.37 ± 0.15
302					-5.10 ± 0.07
304	2.0E-03 ± 1.9E-04	1.44E+07	*	*	
307					-7.87 ± 0.32
310					-1.75 ± 0.15
311					-4.37 ± 0.10
312					-0.37 ± 0.11
313					-2.07 ± 0.08
314					-2.86 ± 0.19
315	1.2E-04 ± 1.3E-05	1.49E+08	1.90E-03 ± 5.31E-05	1.42E+08	
316					-2.23 ± 0.08
317	*	*			-3.53 ± 0.18
318	5.3E-05 ± 1.4E-05	3.26E+07	2.34E-04 ± 1.55E-04	1.17E+07	-1.21 ± 0.30

320	3.7E-04 ± 3.6E-05	5.49E+07	*	*	
321	*	*			
322	2.9E-04 ± 2.0E-05	2.55E+07	†	†	-4.94 ± 0.09
323	*	*			Non-linear
324					Non-linear
326					-5.69 ± 0.14
329					-2.14 ± 0.25
330					-2.79 ± 0.06
331	2.0E-03 ± 5.4E-04	2.08E+06			-3.54 ± 0.07
332					-3.96 ± 0.31
333	stable	stable			-2.89 ± 0.07
334					-3.67 ± 0.09
335	stable	stable	stable	stable	-2.26 ± 0.13
336					-6.36 ± 0.57
337			stable	stable	
339	*	*			-3.48 ± 0.10
